# Virus and CTL dynamics in the extrafollicular and follicular tissue compartments in SIV-infected macaques

**DOI:** 10.1371/journal.pcbi.1006461

**Published:** 2018-10-18

**Authors:** Dominik Wodarz, Pamela J. Skinner, David N. Levy, Elizabeth Connick

**Affiliations:** 1 Department of Ecology and Evolutionary Biology, Department of Mathematics, University of California, Irvine, Irvine, California, United States of America; 2 Department of Veterinary and Biomedical Sciences, University of Minnesota, St. Paul, Minnesota, United States of America; 3 Department of Basic Science, New York University College of Dentistry, New York, New York, United States of America; 4 Division of Infectious Diseases, University of Arizona, Tucson, Arizona, United States of America; ETH Zurich, SWITZERLAND

## Abstract

Data from SIV-infected macaques indicate that virus-specific cytotoxic T lymphocytes (CTL) are mostly present in the extrafollicular (EF) compartment of the lymphoid tissue, with reduced homing to the follicular (F) site. This contributes to the majority of the virus being present in the follicle and represents a barrier to virus control. Using mathematical models, we investigate these dynamics. Two models are analyzed. The first assumes that CTL can only become stimulated and expand in the extrafollicular compartment, with migration accounting for the presence of CTL in the follicle. In the second model, follicular CTL can also undergo antigen-induced expansion. Consistent with experimental data, both models predict increased virus compartmentalization in the presence of stronger CTL responses and lower virus loads, and a more pronounced rise of extrafollicular compared to follicular virus during CD8 cell depletion experiments. The models, however, differ in other aspects. The follicular expansion model results in dynamics that promote the clearance of productive infection in the extrafollicular site, with any productively infected cells found being the result of immigration from the follicle. This is not observed in the model without follicular CTL expansion. The models further predict different consequences of introducing engineered, follicular-homing CTL, which has been proposed as a therapeutic means to improve virus control. Without follicular CTL expansion, this is predicted to result in a reduction of virus load in both compartments. The follicular CTL expansion model, however, makes the counter-intuitive prediction that addition of F-homing CTL not only results in a reduction of follicular virus load, but also in an increase in extrafollicular virus replication. These predictions remain to be experimentally tested, which will be relevant for distinguishing between models and for understanding how therapeutic introduction of F-homing CTL might impact the overall dynamics of the infection.

## Author summary

A better understanding of immune response dynamics and virus control in HIV infection is an important goal of current research. While measurements are often recorded in the blood, intricate dynamics occur in the lymphoid tissue. Recent data indicate that killer T cell responses, or CTL, show reduced homing to the follicular compartment of the lymphoid tissue, while the majority of the CTL remain in the extrafollicular site, which appears to contribute to the observed unequal distribution of virus load in the two locations. Here, these dynamics are studied with 2-compartment mathematical models. They reproduce previously published as well as newly presented experimental data from CTL depletion studies. Beyond this, the models indicate that so far unknown details of the CTL dynamics, in particular the potential of CTL to undergo antigen-induced expansion in the follicular compartment, can be important determinants of outcome. We find that antigen-induced expansion of CTL in the follicular site can result in more pronounced virus compartmentalization, and essentially in clearance of virus-producing cells from the extrafollicular site. We use the models to predict how experimental addition of engineered, follicular-homing CTL to macaques, influence the overall infection dynamics and level of virus control. Understanding these dynamics is an important step in attempts to improve the level of immune-mediated virus control.

## Introduction

Human immunodeficiency virus (HIV-1) causes a persistent infection that eventually progresses to AIDS, following an asymptomatic phase that is variable in duration. Immune responses have been shown to play a major role in determining the level of virus load and the rate of disease progression [1–4]. Cytotoxic T lymphocytes (CTL) are an important branch of the immune system in this respect [1,5,6]. The dynamics between the virus and CTL responses have been documented largely in the blood, and a variety of insights have been obtained about the role of CTL responses for virus control in HIV-infected patients [1,7]. The SIV-infected rhesus macaque model recapitulates many aspects of HIV immunopathogenesis including the induction of virus-specific CTL responses.

The majority of HIV-1 and SIV replication occurs in secondary lymphoid tissues including lymph nodes, spleen, and mucosal associated lymphoid tissues [8–10]. Within secondary lymphoid tissues, the dynamics of virus replication are influenced by the CTL response. CTL are generated in the extrafollicular compartment and display limited homing to the follicular compartment in both HIV-infected humans [11] and SIV-infected rhesus macaques [12]. In SIV-infected macaques with robust CTL responses, virus replication is generally low and the majority of replication is located in the follicular compartment [12]. In contrast, animals with ineffective or absent CTL responses display much more virus replication and an equal distribution of infected cells in the two compartments [12]. These observations give rise to the notion that the presence of effective CTL responses (leading to relatively low virus loads) contributes to the observed unequal distribution of the virus in the two compartments [13].

Here, we investigate these dynamics with the help of two-compartment mathematical models. In agreement with published and newly presented data, the models show that variation in the strength of the CTL response can influence the degree of virus compartmentalization, with stronger CTL responses (that result in lower virus load) correlating with a more unequal distribution of the virus population among the two compartments. Interestingly, details of the outcome of these dynamics can depend on assumptions about CTL activity in the follicular compartment. If it is assumed that CTL can be stimulated and expand to an extent in the follicular compartment, the model predicts a relatively low number of infected cells in the extrafollicular compartment, maintained mostly by immigration from the follicle rather than by successful replication in the extrafollicular compartment itself. If, in contrast, the model assumes that follicular CTL cannot be stimulated to expand, an unequal virus distribution among the compartments still occurs in the model, but higher virus loads are predicted for the extrafollicular compartment, maintained by successful and sustained viral replication in this site rather than by immigration from the follicle. The models further suggest that future experiments involving the addition of follicular-homing CTL to SIV-infected macaques could distinguish between these two assumptions. Without follicular CTL expansion, addition of F-homing CTL is predicted to result in a significant reduction of follicular virus load, and a lesser reduction of extrafollicualr virus load. In contrast, if CTL expansion is allowed to occur, the reduction of follicular virus load upon addition of F-homing CTL is predicted to be accompanied by a significant rise of extrafollicular virus load. A better understanding of these dynamics also has implications for potentially developing the therapeutic use of chimeric antigen receptor T cells that express the follicular homing molecule CXCR5 to improve the overall degree of virus control.

## Results

### A basic two-compartment model without CTL stimulation in the follicular compartment

**Fig 1:**
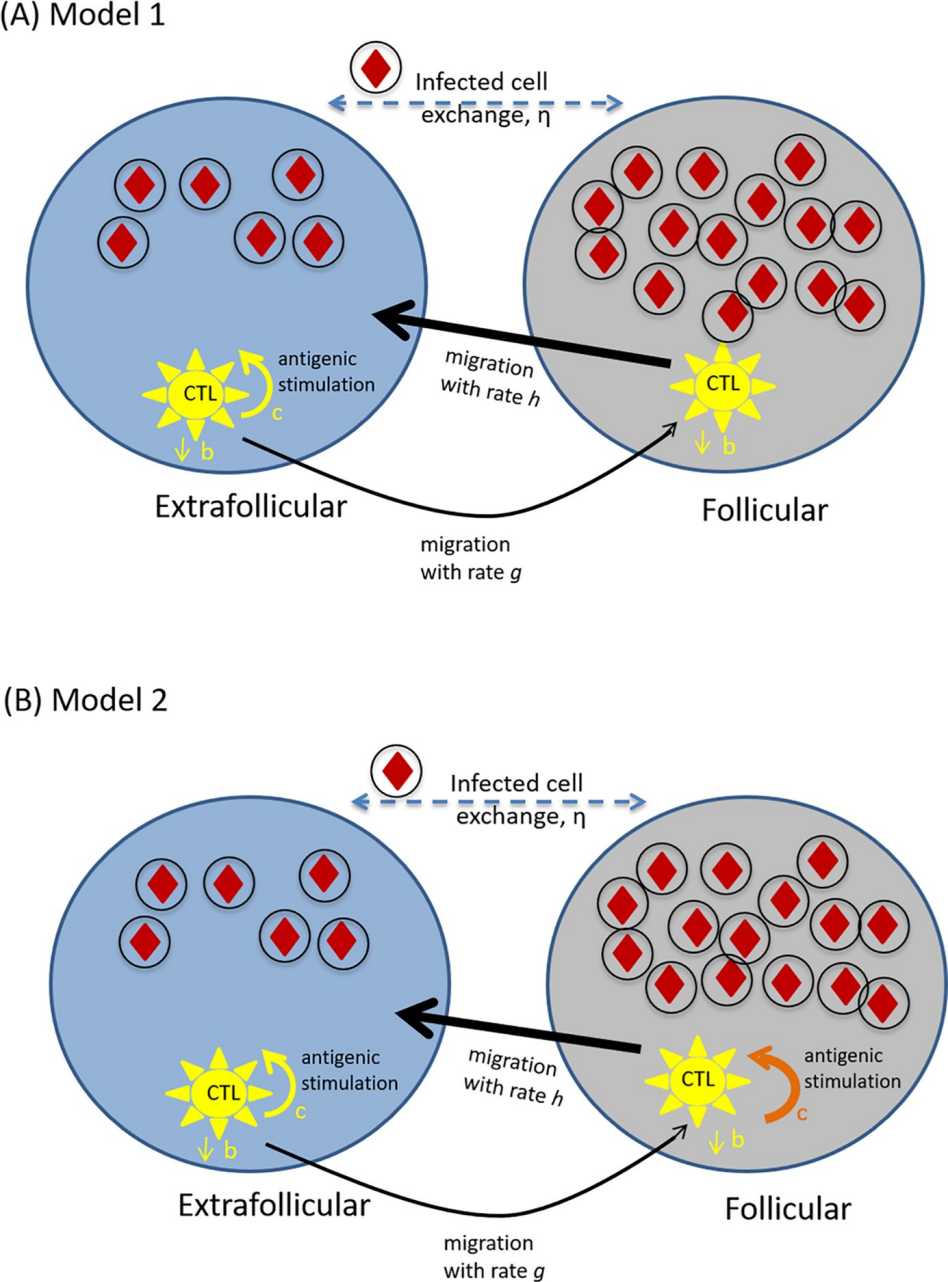
Schematic representation of key model assumptions. (A) Model (1) assuming absence of antigen-induced CTL stimulation and expansion in the follicular compartment. (B) Model (2) assuming that CTL can become stimulated and expand in the follicular compartment. Infected cells are depicted by circles including red diamonds. CTL are shown in yellow. The difference between the two models solely lies in the ability of CTL to expand in the follicular compartment, which is indicated by the absence / presence of the orange expansion arrow in parts A/B.

We consider a two-compartment mathematical model for virus replication (Fig 1A). The virus population can replicate either in the follicular compartment, or in the extrafollicular compartment. The number of uninfected and infected cells in the follicular compartment are denoted by X_f_ and Y_f_, respectively. The corresponding populations in the extrafollicular compartment are denoted by X_e_ and Y_e_, respectively. The main CTL dynamics are assumed to occur in the extralfollicular compartment, and this population is denoted by Z_e_. CTL are also assumed to be able to enter the follicular compartment, and the CTL population there is denoted by Z_f_. The model is thus given by the following set of ordinary differential equations, which describe the average time evolution of the populations.

$$\begin{array}{l} \dot{X_{e}}=\lambda_{e}-dX_{e}-\beta_{e}X_{e}Y_{e}\\ \dot{Y_{e}}=\beta_{e}X_{e}Y_{e}-aY_{e}-pY_{e}Z_{e}-\eta Y_{e}+\eta Y_{f}\\ \dot{X_{f}}=\lambda_{f}-dX_{f}-\beta_{f}X_{f}Y_{f}\\ \dot{Y_{f}}=\beta_{f}X_{f}Y_{f}-aY_{f}-pY_{f}Z_{f}-\eta Y_{f}+\eta Y_{e}\\ \dot{Z_{e}}=cY_{e}-bZ_{e}-gZ_{e}+hZ_{f}\\ \dot{Z_{f}}=gZ_{e}-bZ_{f}-hZ_{f} \end{array}$$ (1)

These equations are an extension of the well-established theoretical framework to mathematically describe the dynamics of virus infections [14–19]. The model does not explicitly take into account the free virus population, which is assumed to be in a quasi-steady state with infected cells (i.e. proportional to the number of infected cells [14]). First, consider the extrafollicular compartment. Uninfected cells are produced with a rate λ_e_, die with a rate d, and upon contact with virus are infected with a rate β_e_. Infected cells die with a rate a, and are killed by CTL with a rate p_e_. The same basic virus dynamics occur in the follicular compartment. Thus, uninfected cells are produced with a rate λ_f_, die with a rate d, and upon contact with virus are infected with a rate β_f_. Infected cells die with a rate a, and are killed by CTL with a rate p_f_. We assume that infected cells can migrate between the two compartments with a rate η. The dynamics of CTL expansion only occur in the extrafollicular compartment. Hence, the CTL expand in response to antigenic stimulation with a rate c. In the absence of stimulation, CTL are assumed to die with a rate b. Extrafolliclar CTL can migrate to the follicular compartment with a rate g. In the follicular compartment, no CTL stimulation / expansion is assumed to occur. CTL can die with a rate b, and they can migrate back to the extrafollicular compartment with a rate h. We note that this model assumes no migration of uninfected cells between the two compartments. A low migration rate would not change the model properties in a meaningful way. If uninfected cells moved between the two compartments with a fast rate, this would affect the relative abundance of the uninfected cells in the two compartments. As mentioned below in Section 5, the distribution of target cells among the two compartments has not been measured, and there is no significant change in conclusions if it is varied within reason. We further note that instead of explicitly tracking the free virus population, we assumed it to be in a quasi-steady state, proportional to the number of infected cells [14]. While it might be possible for free viruses to diffuse more readily between the two compartments than infected cells, fast virus exchange would equalize the distribution of the viruses across the compartments even in the presence of strong immune responses, which is contrary to observations. Hence, the quasi-steady state assumption is likely sufficient for our analysis.

Whether an infection can be established in the absence of immune responses is given by the basic reproductive ratio of the virus [14]. This is defined as the average number of newly infected cells produced by one infected cell during its lifespan when placed into a pool of susceptible cells. It is instructive to consider the situation where there is no virus migration between the two compartments (η = 0), because this significantly simplifies the expressions. In this case, we can define the basic reproductive ratio separately for the follicular and the extrafollicular compartment, given by R_0f_ = λ_f_β_f_/da and R_0e_ = λ_e_β_e_/da. If R_0f_>1, the virus establishes an infection in the follicular compartment. If R_0e_>1, the virus establishes an infection in the extrafollicular compartment. If these conditions are fulfilled, the populations converge to the following equilibrium in the absence of immunity.

$$X_{f}^{*}=\frac{a}{\beta_{f}}\;Y_{f}^{*}=\frac{\lambda_{f}}{a}-\frac{d}{\beta_{f}}\;X_{e}^{*}=\frac{a}{\beta_{e}}\;Y_{e}^{*}=\frac{\lambda_{e}}{a}-\frac{d}{\beta_{e}}$$

Returning to the biologically realistic scenario where infected cell migration occurs between the two compartments (η>0), the virus establishes an infection if the following condition is fulfilled: (*ad*−*β_f_λ_f_*)(*ad*−*β_e_λ_e_*)+*dη*(2*ad*−*β_f_λ_f_*−*β_e_λ_e_*)>0. In this case, the system converges to an equilibrium, the expressions for which are lengthy and uninformative, and therefore not included here. For low values of η, however, the expressions converge to the ones derived for η = 0 above. When a CTL response is added to the system, it reduces virus load towards an equilibrium that is again too lengthy and uninformative to write down. In the mathematical formulation used here, the CTL response always becomes established if the infection persists in the absence of immunity [17] (which is in contrast to other model formulations where establishment of CTL requires a threshold virus load [17]).

In computer simulations, parameter values need to be chosen. Several parameters are unknown (especially for immune responses and compartment-specific processes), and they were chosen arbitrarily for the purpose of illustration. The presented simulation results are not dependent on this particular choice of parameter values unless otherwise stated (see section 5 for more in depth discussion of parameter effects). Known parameters are adopted from the literature. Hence, infected cells are assumed to have an average life-span of around 2.2 days [20], and other viral replication parameters were adjusted such that the basic reproductive ratio of the virus was approximately eight [21–23]. In the following, we examine how immune parameters determine virus load in the two compartments. We will thereby concentrate on two parameters. The first is the strength of the CTL response, defined as the rate at which CTL respond to antigen, or CTL responsiveness c. This parameter captures the many biological processes that contribute to the rate of activation and expansion when a CTL is exposed to antigenic stimulation. The second parameter is the rate of CTL migration from the extrafollicular to the follicular compartment, g. While we concentrate on those parameters, the effect of varying other parameters, as well as scaling effects, are described in Section 5.

#### Variation in the CTL responsiveness, c

**Fig 2:**
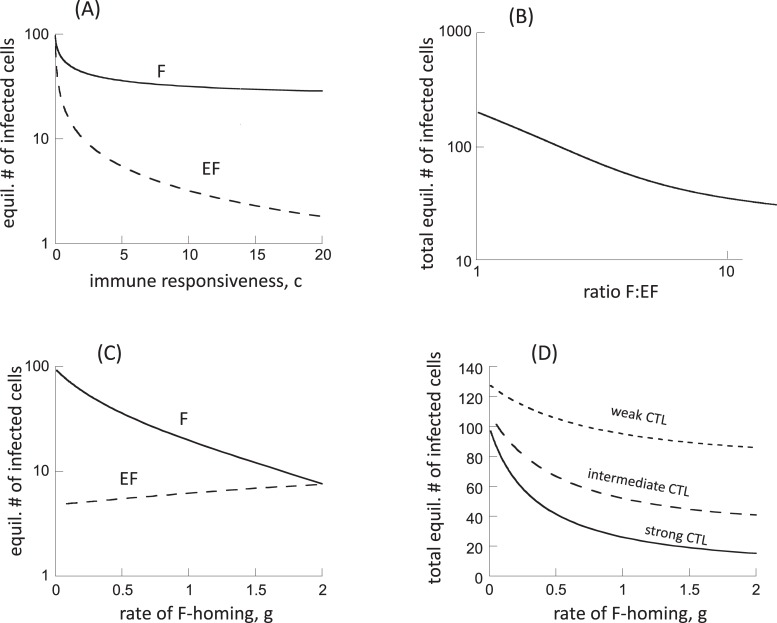
Properties of model (1), assuming absence of antigen-induced stimulation in the follicular compartment. (A) Equilibrium number of virus-producing cells in the two compartments as a function of the CTL responsiveness, c, which correlates with the strength of the CTL response. F = follicular compartment, EF = extrafollicular compartment. (B) Relationship between the equilibrium number of virus-producing cells and the ratio of follicular to extrafollicular virus load (F:EF). (C) Equilibrium number of virus-producing cells in the two compartments as a function of the rate at which CTL home to the follicular compartment, g. We note that the graph does not start at g = 0, but at g = 0.01. (D) Total equilibrium number of virus-producing cells (summed over both compartments) as a function of the rate at which CTL home to the follicular compartment, g. Base parameters were chosen as follows. λ_e_ = λ_f_ = 50, d = 0.01, β_e_ = , β_f_ = 0.00072, a = 0.45, η = 0.01, c_e_ = 5, b = 0.01, p = 0.001, g = 0.5, h = 2. For (D), we assumed c_e_ = 5 for the strong CTL response, c_e_ = 1 for the intermediate response, and c_e_ = 0.2 for the weak response.

In the mathematical model considered, the CTL responsiveness parameter, c, is one of the most important immune parameters that determines virus load at equilibrium [24]. Thus, a lower value of c results in higher virus load, which can correspond to advanced stages of the disease when immune responses have been weakened. Similar considerations could apply to the very early stages of the disease, before immune responses have fully developed, although this does not represent any kind of equilibrium situation. A higher value of c would correspond to the early chronic phase of the infection, especially in good controllers, when virus load is relatively low. In the following, we assume that virus infection parameters are the same in both compartments. Fig 2A plots the equilibrium number of infected cells in the two compartments, as a function of the CTL responsiveness, c. An increase in the parameter c results in overall lower virus loads. The decline in virus load is more pronounced in the extrafollicular compared to the follicular compartment (Fig 2A). For very large values of c, the equilibrium number of extrafollicular infected cells tends towards zero, while the equilibrium number of follicular infected cells tends towards a constant. These trends is also reflected in Fig 2B, showing a negative association between total virus load and the ratio of follicular: extrafollicular (F:EF) virus load, as the rate of CTL expansion is varied. That is, the lower the overall number of infected cells, the stronger the degree of compartmentalization observed in the model. For the highest virus loads in the model (weak CTL), the distribution of the infected cell populations in the two compartments becomes equal for the chosen parameters (F:EF ratio converges to 1, Fig 2A and 2B). This is consistent with experimental data that show a dominance of the virus population in the follicular compartment in the chronic phase of SIV infection, when CTL responses are strong, and a more equal distribution during advanced disease or in the very early stages of infection, when CTL-mediated suppression of the virus is weaker [12].

#### Variation in the rate of CTL homing to the follicular compartment, g

The rate at which CTL move from the extrafollicular to the follicular compartment (g) can also influence the distribution of virus in the two compartments in the model. It is unclear whether this parameter is biologically fixed or whether it can be influenced by the virus. Varying this parameter in the model, however, allows us to study how it can potentially affect infection outcome. At relatively low CTL F-homing rates, the virus is more abundant in the F compared to the EF compartment (Fig 2C). If more CTL move from EF to F (larger value of g), virus load in the F compartment decreases significantly, because more CTL-mediated killing occurs in the F compartment (Fig 2C). At the same time, virus load in the EF compartment rises a little (Fig 2C), because homing of CTL to the follicular compartment leaves fewer CTL in the extrafollicular compartment. This rise, however, is not very pronounced. If the homing rate of CTL from the extrafollicular to the follicular compartment becomes equal to the movement of CTL back from the F to the EF compartment (g = 2, Fig 2C), the distribution of the virus population across the two compartments becomes even for the parameter combinations considered. Summed over both compartments, total virus load declines with increased homing of CTL to the F compartment (Fig 2D). The extent of this decline, however, depends on the strength of the CTL response (parameter c). The stronger the CTL response, the more pronounced this effect (Fig 2D).

### Model with CTL stimulation in the follicular compartment

Here, the above analysis is repeated, assuming that CTL can also be stimulated and expand in the follicular compartment (Fig 1B). The equations for the CTL dynamics are thus given by

$$\begin{array}{l} \dot{Z_{e}}=c_{e}Y_{e}-bZ_{e}-gZ_{e}+hZ_{f}\\ \dot{Z_{f}}=c_{f}Y_{f}-bZ_{f}+gZ_{e}-hZ_{f} \end{array}$$ (2)

The equations for the virus dynamics remain the same as in model (1). The CTL are now assumed to expand in both compartments, with rates c_e_ and c_f_, respectively. Since we currently do not have any information about the relative magnitude of these parameters, we will assume c = c_e_ = c_f_ for purposes of illustration. Results, however, do not depend on this particular choice (see section 5). Although CTL in model (2) can expand in response to antigenic stimulation in the follicular compartment, the rate at which they migrate back to the extrafollicular compartment (h) is set to be large relative to the rate of entry into the follicular compartment (g). The effect of this is that while stimulation does occur in the follicle, most of the cells that arise from this expansion quickly re-enter the extrafollicular compartment, thus limiting the number of CTL that remain in the follicle. In the absence of this assumption, follicular CTL expansion would result in accumulation of CTL at this location and would thus be unrealistic.

**Fig 3:**
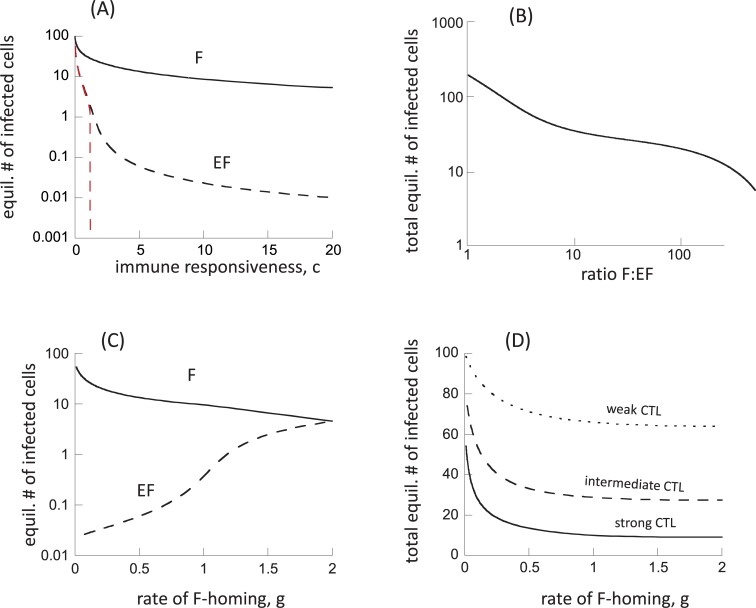
Properties of model (2), assuming the presence of antigen-induced stimulation in the follicular compartment. (A) Equilibrium number of virus-producing cells in the two compartments as a function of the rate of CTL expansion. Since CTL are assumed to expand in both compartments in this model, we varied the expansion rate in both, by setting c = c_e_ = c_f_. The red dashed lined depicts results under the assumption that virus-producing cells cannot migrate between the two comparmtents, η = 0. (B) Relationship between the equilibrium number of virus-producing cells and the ratio of follicular to extrafollicular virus load (F:EF). (C) Equilibrium number of virus-producing cells in the two compartments as a function of the rate at which CTL home to the follicular compartment, g. We note that the graph does not start at g = 0, but at g = 0.01. (D) Total equilibrium number of virus-producing cells (summed over both compartments) as a function of the rate at which CTL home to the follicular compartment, g. Base parameters were chosen as follows. λ_e_ = λ_f_ = 50, d = 0.01, β_e_ = , β_f_ = 0.00072, a = 0.45, η = 0.01, c_e_ = c_f_ = 5, b = 0.01, p = 0.001, g = 0.5, h = 2. For (D), we assumed c_e_ = 5 for the strong CTL response, c_e_ = 1 for the intermediate response, and c_e_ = 0.2 for the weak response.

While this model generally has similar properties as model (1), there are some significant differences that arise from the assumption that CTL can be stimulated in the follicular compartment (and subsequently re-enter the EF compartment to exert immune pressure there). This introduces an element of “indirect” or “apparent” competition among viruses in the different locations [25,26], mediated by a shared CTL response. In model (1), the dynamics in the extrafollicular compartment were governed by the interactions between the local virus population and the CTL population that was stimulated in that compartment only. In model (2), however, CTL are independently added to the extrafollicular compartment by immigration, following antigenic stimulation in the follicular compartment. This increases immunological pressure on the extrafollicuclar virus population. As a consequence, equilibrium virus load in the extrafollicular compartment is predicted to decline to a larger extent if the strength of the CTL response is increased, compared to model (1), see Fig 3A (we varied the rate of CTL expansion in both compartments, such that c = c_e_ = c_f_). Similar to model (1), an increase in the F:EF ratio is observed as virus load becomes lower (Fig 3B). For the same parameter ranges as in model (1), however, we observe higher F:EF ratios in model (2) (Fig 3B). The reason is the more pronounced reduction in the equilibrium number of extrafollicular infected cells with stronger CTL responses, which leads to more extensive compartmentalization. The exact values of the F:EF ratios depends on the choices of parameter values, which are currently unknown.

It is instructive to also plot the extrafollicular number of infected cells at equilibrium under the assumption that infected cells do not migrate between the two compartments (η = 0, dashed red line in Fig 3A). Now, the extrafollicular number of virus-producing cells goes extinct if the strength of the CTL response, c, crosses a threshold. The reason is that the CTL that are stimulated in the F compartment and move to the EF compartment put extra pressure on the virus population in the EF compartment, thus clearing the virus in this site. In the presence of infected cell migration (η>0, dashed black line in Fig 3A), the persistence of extrafollicular virus beyond this CTL strength threshold is the result of infected cell migration from the follicular to the extrafollicular compartment, and not maintained by active virus replication in the EF compartment itself.

Some differences are also observed when varying the rate at which CTL home from the extrafollicular to the follicular compartment (parameter g, Fig 3C). If the rate of CTL homing to F is low (low g), there is relatively high virus load in the F compartment, and very few infected cells are present in the EF compartment, only due to immigration of infected cells from the follicular compartment, as described above. For higher rates of CTL homing to the F compartment, follicular virus is reduced because more CTL enter this compartment and kill infected cells. At the same time, virus load in the EF compartment rises significantly (much more than in model (1)), and is now maintained by ongoing productive infection in the EF compartment (rather than just by immigration). The reason is two-fold. (i) As in model (1), a larger number of the CTL leave the EF compartment and enter the F compartment, resulting in less killing of infected cells in the EF compartment. (ii) In addition, and more importantly, lower follicular virus load results in less stimulation of follicular CTL that can migrate back to the EF site, thus reducing external CTL-mediated pressure on EF virus. For large values of g that are comparable to the rate of CTL migration back to the EF compartment, h, the virus in the two compartments becomes equally distributed. While increased CTL homing to the follicular compartment is predicted to result in an increase in EF virus load and a decline in follicular virus load, total virus load (the sum of the virus population in F and EF compartments) is predicted to decline to a certain extent (Fig 3D). As in model (1), this decline becomes less pronounced for weaker CTL responses (Fig 3D).

### Predictions about experimental manipulations

In the above sections, we have shown how the dynamics of CTL responses can influence the distribution of virus load across the two compartments. Hence, experimental manipulation of the CTL responses, such as CTL depletion or addition, should result in changes in these distributions. We have considered two mathematical models, one assuming that CTL cannot be stimulated to proliferate in the follicular compartment, while the second model did allow for CTL stimulation in this site. Here, we explore how CTL depletion and addition experiments are predicted by the two models to change the virus distribution in the two compartments, and how these predictions compare to experimental data. We will explore whether the comparison between models and data can be used to distinguish between the two models considered.

#### Simulating CTL depletion experiments

**Fig 4:**
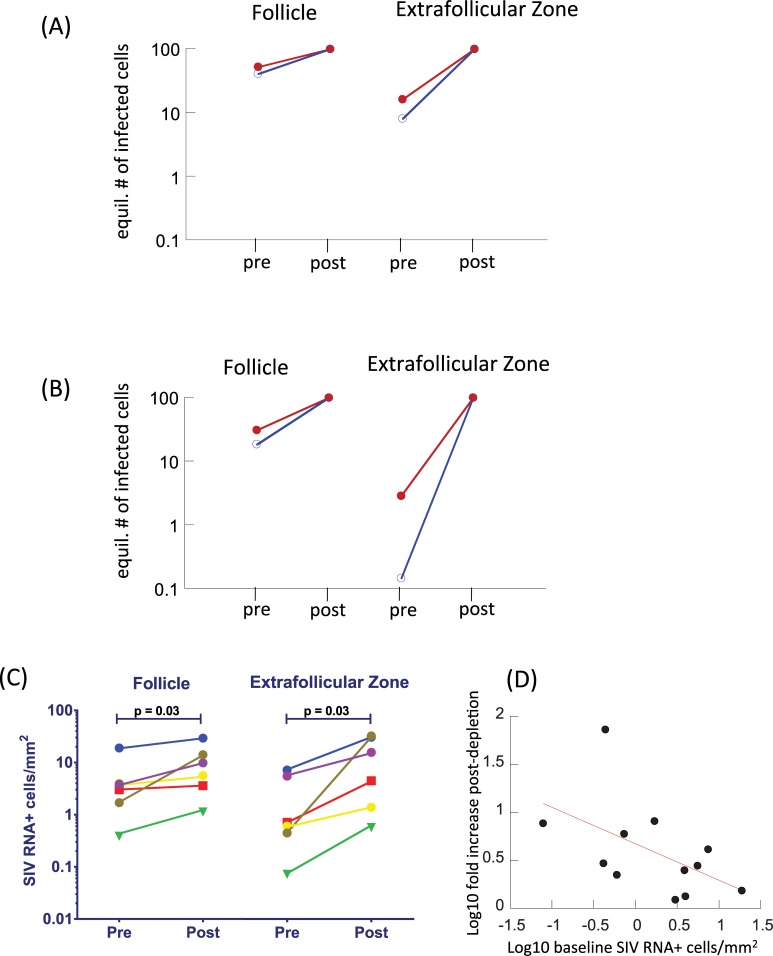
**(A,B) Simulation of CTL depletion experiments with (A) model (1) assuming no antigen-induced CTL expansion in the follicular compartment, and (B) model (2) assuming the presence of antigen-induced CTL expansion in the follicular compartment.** The equilibrium number of virus-producing cells is plotted in the presence of CTL (pre) and in their absence (post). This was done for a weaker CTL response / higher baseline virus load (red) and a stronger CTL response / lower baseline virus load (blue). Parameters were chosen as follows. λ_e_ = λ_f_ = 50, d = 0.01, β_e_ = β_f_ = 0.00072, a = 0.45, η = 0.01, b = 0.01, p = 0.001, g = 0.5, h = 2. For the weaker CTL response, c_e_ = 1 (and c_f_ = 1 for model 2), for the stronger CTL response c_e_ = 3 (and c_f_ = 3 for model 2). (C) Results from CD8 cell depletion experiments, performed in SIV-infected rhesus macaques, showing SIV RNA levels pre and 14 days post administration of an anti-CD8 antibody. The different colors represent data from different experimental animals. See text for details. The data are based on experiments generated for a previous study [27], but contain so far unpublished data. CD8 cell depletion results in a significant increase in the number of SIV RNA+ cells in both compartments, as shown. The fold-increase is significantly larger in the EF compared to the F compartment (p = 0.03, Wilcoxon test). (D) Correlation between baseline SIV RNA levels pre depletion, and the fold-increase of SIV RNA levels post depletion. Data from both compartments are included, showing a significant negative correlation (p = 0.04), as suggested by the mathematical models.

Here we show model predictions about the outcome of experiments in which CTL are depleted from SIV-infected macaques, and compare predictions to experimental data. To do so, we compare the equilibrium number of infected cells in the presence of CTL (corresponding to pre-depletion), and in the absence of CTL (corresponding to post-depletion, Fig 4). This was done for both model (1) (Fig 4A) and model (2) (Fig 4B). The trends are qualitatively similar for both models. CTL depletion is predicted to result in an increase in the number of virus-producing cells, with the increase being more pronounced in the extrafollicular compared to the follicular compartment. More generally, the rise is more pronounced if the baseline number of virus-producing cells is lower due to a stronger CTL response. The increase in the number of virus-producing cells in the extrafollicular compartment is predicted to be stronger for model 2 (Fig 4A and 4B), because suppression of EF virus load is stronger for model (2) compared to model (1).

These predicted patterns are also seen in the experimental data. Although comparison to data cannot be used to distinguish between the two models, it does establish that the modeling approaches used here are consistent with available experimental information. We evaluated the impact of CD8+ cell depletion on the frequency of virus-producing cells within follicular and extrafollicular compartments in lymph nodes from 6 chronically SIV-infected rhesus macques as previously described [27]. Prior to CD8+ cell depletion, all animals demonstrated higher concentrations of virus-producing cells within the follicles than in the extrafollicular compartments. While in the current data, this difference does not reach statistical significance due to small sample size, a convincing statistical difference has been demonstrated in previous studies, both in rhesus macaques [12] and in humans [11,28]. Fourteen days after administration of an anti-CD8 antibody, frequenices of virus-producing cells increased in both compartments, and this increase was significantly more pronounced in the extrafollicular compartment, such that similar levels were found in both compartments. Notably, animals with the lowest frequencies of virus-producing cells at baseline demonstrated the largest increases in virus replication in both compartments, whereas those with the highest frequencies of virus-producing cells at baseline demonstrated more muted responses to CD8 depletion (Fig 4C and 4D). These findings further underscore the hypothesis that the CTL response is a major contributor to the distribution of virus within the lymph node.

#### CTL addition experiments

To address the deficiency of virus-specific CTL in B cell follicles and reduce virus replication at those sites, we have proposed to introduce the follicular homing molecule CXCR5 into virus-specific CTL [29]. Binding of CXCR5 by its ligand CXCL13 does not induce proliferation of T cells. It induces chemotaxis of the cells towards higher concentrations of CXCL13, which are in the follicle. It is possible that when the CTL contact virus-producing cells in the follicle, they will be stimulated to proliferate by engagement of the T cell receptor.

**Fig 5:**
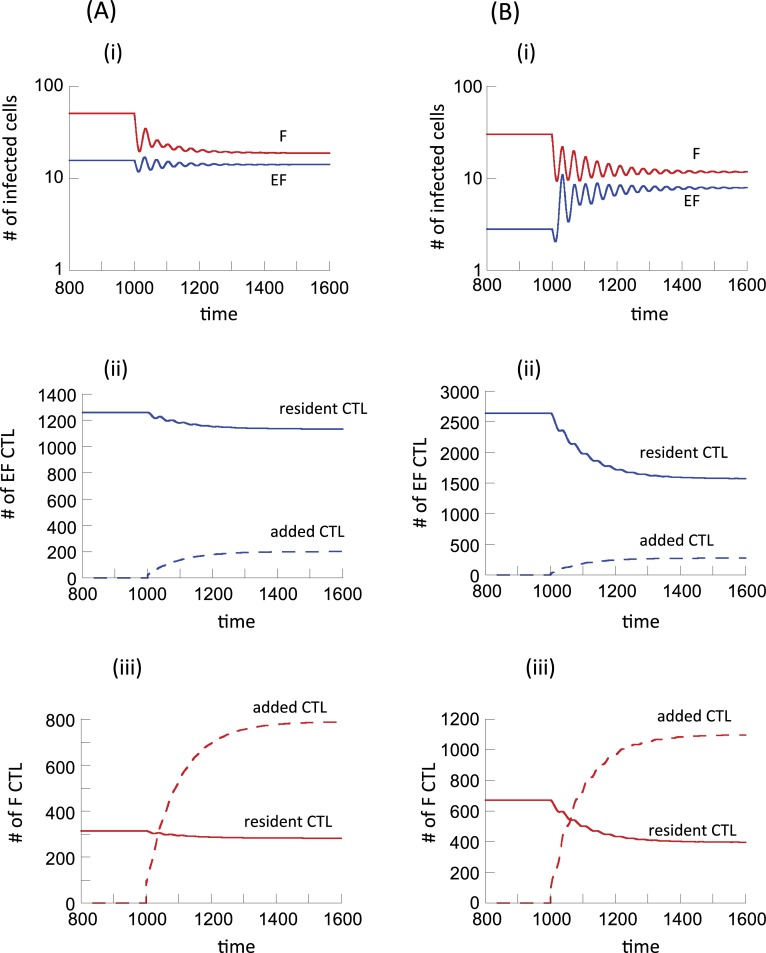
**Simulation of experiments where CTL that preferentially home to the follicular compartment are added to a system at equilibrium, using (A) model (1) assuming no antigen-induced CTL expansion in the follicular compartment, and (B) model (2) assuming the presence of antigen-induced CTL expansion in the follicular compartment.** The system was first allowed to equilibrate, and at time step 1000, one hundred F-homing CTL were added to the F compartment. Resident and F-homing CTL were tracked as separate populations, as described in the text. Both part A (left side) and part B (right side) have panels i-iii. Shown are the dynamics of virus-producing cells in the two compartments (panel i), the CTL dynamics in the EF compartment (panel ii), and the CTL dynamics in the F compartment (panel iii). Parameter values were chosen as follows. λ_e_ = λ_f_ = 50, d = 0.01, β_e_ = , β_f_ = 0.00072, a = 0.45, η = 0.01, c_e_ = c_f_ = 1, b = 0.01, p = 0.001. For the resident CTL, g = 0.5, h = 2. For the added F-homing CTL, g = 2, h = 0.5. Further, we assumed a lower expansion rate for the added CTL, given by c2_e_ = 0.7 and also c2_f_ = 0.7 for model (2).

We simulated potential experiments where F-homing CTL are added to experimental animals. To do so, we considered a second population of CTL with F-homing characteristics and added those to the F compartment where cell populations were at equilibrium (details given in the legend to Fig 5). In this model, resident and added CTL were tracked as two distinct populations. The analysis was done for model (1) (Fig 5A), and for model (2) (Fig 5B). In terms of model parameters, the resident and added CTL populations differed as follows. The resident CTL were assumed to have a low migration rate from EF to F, but a high migration rate from F back to EF, as has been assumed so far. This results in the majority of the CTL residing in the extrafollicular compartment. The added CTL population has the opposite characteristics because they are supposed to be F-homing. They have a fast migration rate from EF to F, and a slow migration rate from F back to EF. Hence, the added CTL tend to accumulate in the F compartment. It was further assumed that the antigen-induced proliferation rate of the added CTL was somewhat lower than that of the resident CTL. The rationale behind this assumption is that experimental addition of CTL might result in a reduction in their efficacy compared to native CTL, although model results do not depend on this assumption.

For Model 1, the simulation results are shown in Fig 5Ai. CTL addition results in a decline in the number of virus-producing cells in both compartments, with the decline being more pronounced in the follicular compartment. The virus-producing cell population declines only slightly in the extrafollicular compartment (which might be difficult to measure experimentally) because some of the added F-homing CTL enter the extrafollicular compartment through migration, resulting in additional killing there. This is also reflected in Fig 5Aii-iii, showing the dynamics of resident and added CTL in both compartments. The added F-homing CTL become dominant in the follicular compartment, and make up a minority population in the extrafollicular compartment.

The same analysis was performed for model (2) assuming that antigen-induced CTL expansion can occur in the follicular compartment. Now, slightly different behavior is observed (Fig 5Bi). As before, follicular virus load is reduced upon addition of the F-homing CTL. At the same time, however, extrafollicular virus load increases, which is the opposite compared to model (1). The reason is that in model (2), follicular virus stimulates CTL to proliferate, and the resident CTL resulting from this proliferation migrate back to the EF compartment with a fast rate, suppressing EF virus load. When F-homing CTL are added, this force is reduced. F-homing CTL reduce virus load in the follicular compartment and become dominant relative to the resident CTL in this site (Fig 5iii). Therefore, stimulation and subsequent migration of resident CTL back to the EF compartment occurs with a reduced rate, allowing the extrafollicular virus population to replicate at higher levels.

These predictions show that experiments involving the addition of F-homing CTL can help distinguish between the two models, based on the virus dynamics in the extrafollicular compartment that occurs upon addition of the F- homing CTL. While such experiments have not yet been reported, they are an important next step to perform. This knowledge will further be important to evaluate the use of F-homing CTL as a potential therapeutic strategy in order to reduce the relatively uncontrolled follicular virus population. If this indeed results in a rise in extrafolliuclar virus load, additional approaches would have to be employed to address this issue.

### Parameters and assumptions

The analysis concentrated on the effect of two key parameters: The rate of CTL expansion, c, and the rate at which CTL move from the extrafollicular to the follicular compartment, g.

In the literature, the rate of CTL expansion has often been used as a key parameter that determines the extent of CTL-mediated virus control. Other parameters, however, also drive virus load in our models. In the model formulations considered here, the rate of CTL-mediated killing, p, has the same effect as the rate of CTL expansion, c, such that the equilibrium virus load is determined by the product c*p. Therefore, the effect of the parameter p on the degree of virus compartmentalization is the same as that of the parameter c. Another important determinant of virus control is the death rate of CTL, b, with lower values of b resulting in lower equilibrium virus loads. Therefore, lower values of b result in more virus compartmentalization, as shown in S1 Text.

In the context of CTL migration processes, we varied the rate at which CTL move from the extrafollicular to the follicular compartment (parameter g) and kept the rate of back-migration from F to EF (parameter h) constant. The distribution of CTL can, however, also be influenced by the parameter h. In fact, if the parameter g is sufficiently large, an increase in g has an identical effect as an equivalent decrease in h, and it is the ratio of g/h that counts (S1 Text). For lower value of g, however, the number of CTL that enter the F compartment is limited. Hence, in this parameter regime, an increase in the parameter g does not have a quantitatively identical effect compared to an equivalent decrease in h, and we need to consider the dependence of the outcome on the individual parameters rather than on the ratio of g/h. This is illustrated with computer simulations in S1 Text.

In the simulations presented in the figures, parameter values were chosen such that the migration rates of infected cells were generally slower than those of CTL among the two compartments. As far as we are aware, there are currently no data available that would allow us to estimate the respective migration rates. The results presented in the figures, however, largely do not depend on those parameter choices (see S1 Text). If the migration rate of infected cells is sufficiently large, the degree of virus compartmentalization is reduced because the system essentially starts behaving more like a single well-mixed compartment, which is not a parameter region of interest in the current study.

Due to lack of appropriate data, it is further not clear how the number of target cells compares in the two compartments, and how cell parameters differ between the two compartments. For simulations, we chose parameters such that the number of susceptible target cells in the absence of infection is the same for the two compartments, but the results presented here do not depend on this choice of parameters. In S1 Text, we show that simulation results remain qualitatively the same if there are significant differences in target cell parameters and target cell numbers in the two compartments, brought about by differences in parameters λ_e_ and λ_f_. Results further remain robust if we assume asymmetrical migration rates of the target cells between the two compartments, and if CTL expansion occurs at different rates in the two compartments (S1 Text).

## Discussion

Experiments with SIV-infected macaques indicated that in chronic infection, there is an unequal distribution of virus in the follicular and extrafollicular compartment of lymphoid tissues [12,13]. The majority of the virus population was shown to reside in the follicular compartment. Data further indicate that CTL have a reduced ability to home to the follicular compartment, which can contribute to the observed virus compartmentalization. Since the differential CTL activity can contribute to the unequal virus distribution, the degree of virus compartmentalization is highest for strong CTL-mediated virus control (leading to low virus loads), and the distribution of the virus among the compartments becomes more equal for less efficient CTL-mediated virus control (higher virus loads) [12,13]. We used mathematical models to investigate in more detail how the dynamics of CTL responses can affect the distribution of the virus population across the two compartments, and how different assumptions about the CTL responses can influence outcome. In particular, there remains considerable uncertainty about CTL-related processes that occur in the follicular compartment. Data indicate that a relatively small population of CTL does enter the follicular compartment, and that these CTL can reduce virus load to a certain extent in this site [27]. It is unclear whether virus-producing cells in the follicular compartment are able to induce proliferation of the CTL, or whether expansion processes are absent in this compartment, and if so why. Therefore, we compared the properties of two models, one in which no CTL expansion was allowed to occur in the follicular compartment, and one in which expansion processes did take place there, with subsequent migration of the expanded CTL back to the extrafollicular compartment. The predictions of both models agreed with experimental data that are available so far. Thus, as seen in data, both models predict an increasing ratio of F:EF virus load with stronger CTL responses and lower equilibrium virus loads. In other words, while significant virus compartmentalization occurs in the model for strong CTL responses and low virus loads, virus distribution is predicted to be significantly more equal for weaker CTL responses and higher virus loads. Similarly, simulation of CTL depletion experiments with both models predicts that depletion results in a rise of virus load in both compartments, and that this rise is more pronounced in the extrafollicular compared to the follicular compartment, consistent with our experimental observations shown here and reported previously [27]. These two predictions are actually not independent of each other, as they both depend on the strength of the CTL response, and the models considered here might not be the only ones with these properties. The match of these predictions with experimental data, however, means that both model (1) and model (2) are consistent with available data, and hence are for now appropriate to explore.

While both models are consistent with available experimental observations, they significantly differ in other aspects. In particular, the degree of compartmentalization is predicted to be stronger for model (2) with follicular CTL proliferation compared to the model (1) without. In fact, in model (2), a possible outcome is the clearance of extrafollicular productive infection, were any virus-producing cells found in the extrafollicular compartment are the result of immigration from the follicle. There is experimental indication that virus-producing cells found in the EF compartment are indeed the result of immigration from the F compartment rather than the result of sustained extrafollicular virus replication, supporting the prediction of model (2) that productive infection in the EF compartment is essentially cleared by CTL: In the gut of SIV-infected macaques, it was observed that virus-producing cells in the extrafollicular region tended to be located near the follicles, in fact adjacent to them [12], indicating that they have recently immigrated from there. Additionally, this model-predicted outcome is supported by the data from well-controlled SIV infection, in which effective clearance of productive infection in the extrafollicular compartment was reported [12,30]. It is intriguing that Ki67+ virus-specific CTL are detected in follicles [27], adding to the data that support follicular CTL expansion as a physiologically relevant process, as assumed in model (2). In model (1) without follicular CTL expansion, on the other hand, there is no equilibrium or outcome that corresponds to clearance of extrafollicular productive infection. If the CTL response is very strong, the number of productively infected cells predicted by the model can in theory be sufficiently low to essentially correspond to clearance in a stochastic setting. Whether this is a likely scenario, however, is unclear, especially given data that document significant functional impairment of HIV/SIV specific CD4 T helper cell responses [31], which in turn negatively impact the effectiveness of CTL responses [5,32].

Our analysis indicates that experiments involving the addition of follicular-homing CTL to SIV-infected macaques could help to further distinguish between the two models considered here, based on the dynamics of virus-producing cells in the extrafollicular compartment following the addition of those CTL. If a significant increase of extrafollicular virus load occurs after CTL addition, CTL stimulation is likely to occur in the F compartment, as mathematically described in model (2). In contrast, if the addition of follicular-homing CTL does not result in significant changes in the number of extrafollicular virus-producing cells, we can discard the assumption that CTL can become stimulated in the follicle. If this is the case it is possible that other, yet unknown, mechanisms need to be invoked to explain the observed compartmentalization patterns and the effective clearance of productive infection in the extrafollicular compartment.

The use of chimeric antigen receptor T cells that express the follicular homing molecule CXCR5 could be an interesting therapeutic option to reduce virus load in the follicular compartment and hence to improve the overall degree of virus control. This could be a component of treatment approaches that aim for a cure. In this context, it will be important to further address the dynamics suggested by our models, in particular whether improved follicular virus control mediated by follicular homing CTL might lead to a concomitant increase in extrafollicular virus load as suggested by model (2).

As with any model, the results can depend on assumptions and the particular way in which the model is formulated. The biggest uncertainty of such models is the way in which the CTL response is formulated [17,33,34]. Many models used in the literature assume that the rate of CTL expansion is proportional not only to the number of virus-producing cells, but also to the number of CTL that are present. The dynamics in such models, however, tend to be rather unstable, involving pronounced and prolonged oscillations in CTL and virus populations, which are not typically observed in vivo. We chose a model where the rate of CTL expansion was only proportional to the number of virus-producing cells, because such models have been shown previously to display more realistic and stable behaviors that are more consistent with observations [17]. Nevertheless, computer simulations indicate that the main results reported here do not depend on these particular details.

Another aspect to note is that while the models considered here are consistent with currently available experimental data, this does not mean that the models are necessarily correct. There could be other models, with alternative assumptions that we have not thought of, that are just as consistent with the available experimental data. On the flip side, if a mathematical model is not consistent with data, we can discard the underlying assumptions with some confidence. For example, one mechanism that we hypothesized might independently drive infected cell compartmentalization is a difference in the activation status of CD4 T cells in the two compartments. While follicular CD4 T cells are by default activated and highly permissive to infection [35], the activation status and thus permissiveness of extrafollicular CD4 T cells might vary, and might be driven by the amount of virus present in the EF compartment [36]. We formulated such a process mathematically in S1 Text, and concluded that this mechanism alone does not result in dynamics that are consistent with data available so far.

While there are uncertainties about particular aspects of the models, they clearly demonstrate how differences in assumptions about the mechanisms of CTL activity in the two compartments can lead to different dynamics. This in turn can help to interpret future experimental data, to potentially exclude certain hypotheses about CTL activity in the follicular compartment, and to sharpen our thinking about how potential therapeutic efforts to increase CTL-mediated activity in the follicular compartment could impact the overall dynamics of the infection.

## Methods

The majority of this paper is computational in nature. The mathematical models and the analysis are fully presented in Results section as well as in S1 Text. In addition to the computational analysis, experimental data from CD8 cell depletion experiments in SIV-infected macaques are presented. These data were generated in the context of a previous study, according to the same methodology described in [27]. Procedures are briefly outlined in the manuscript text. The data shown in figures have, however, not been previously published.

## Supporting information

S1 Text Presentation of additional computational details that are not provided in the main text. In particular, details are given about a mathematical model version in which virus-induced target cell activation promotes permissiveness of the cells to infection.
(PDF)
