# Supplementary material for: Virus and CTL dynamics in the extrafollicular and follicular tissue compartments in SIV-infected macaques
Source: PLoS Comput Biol. 2018 Oct 18;14(10):e1006461. doi: 10.1371/journal.pcbi.1006461 (PMC6207320; doi:10.1371/journal.pcbi.1006461)
Supplement: S1 Text — In particular, details are given about a mathematical model version in which virus-induced target cell activation promotes permissiveness of the cells to infection. (PDF) [file pcbi.1006461.s001.pdf]

Supplementary Text S1

Parameter dependencies and scaling

The analysis presented in the main text concentrated mainly on varying two parameters: The parameter c, the rate of CTL expansion, was varied because it is a major determinant of the equilibrium number of infected cells, and hence of the degree of CTL-mediated virus control. It is therefore a measure of the CTL "strength". The parameter g was varied because we wanted to explore the effect of changing the ability of the CTL to home to the follicular compartment. Besides the parameters that we varied in the main part of our analysis, however, other parameters also influence the ability of CTL to reduce virus load, or the tendency of the CTL to accumulate in the follicular compartment. In section 5 of the main text, we discuss the effects of those alternative parameters. In the current section, we present select simulation results to support the arguments in the main text. Further, due to lack of estimates for some of the parameter values, arbitrary assumptions were made. Thus, we assumed that the number of susceptible target cells was identical in the two compartments, and that the movement rate of infected cells from the follicular to the extrafollicular compartment is the same as that for the opposite direction. In section 5 of the main text, we re-visited predictions in parameter regimes where target cell kinetics differ in the two compartments, and where movement rates between the two compartments are asymmetric. Supporting computer simulations are shown in the current section.

Varying the degree of CTL-mediated virus control: As mentioned in the main text, besides the rate of CTL expansion, c, the equilibrium number of infected cells is also determined significantly by other CTL parameters, such as the rate of CTL-mediated lysis, p, and the death rate of CTL. b. In fact, the parameter p has an identical effect on the equilibrium number of infected cells as the parameter c. That is, a given increase the parameter c results in a reduction in the equilibrium number of infected cells that is identical to that observed for the same increase in the parameter p. Therefore, these two parameters are interchangeable in the context of the model we investigated here.

Due to unknown immune parameter values, the figures shown in the main text for model (2) assume identical CTL expansion rates for the extrafollicular and follicular compartments, i.e. $c_e=c_f$. Here, we show that results remain similar if we assume different CTL expansion rates in the two compartments. In Figure A, the graph from Figure 3A in the main text is replotted in black ($c_f = c_e$), in addition to an equivalent graph assuming that $c_f = 0.5c_e$ (Red). This demonstrates that the general patterns remain qualitatively the same.

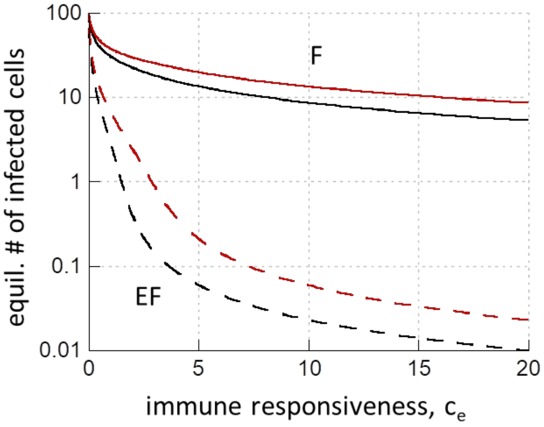

Figure A: Equilibrium number of virus-producing cells in the two compartments as a function of the immune responsiveness c_e. The black lines show the same graph as in Figure 3A in the main text ($c_f=c_e$), and the red lines are new, assuming $c_f=0.5c_e$. F=follicular compartment (solid lines), EF=extrafollicular compartment (dashed lines). Parameters are the same as in Figure 3A in the main text.

The CTL death rate, b, is also an important determinant of the number of infected cells at equilibrium, with lower values of b (longer lived CTL) resulting in fewer infected cells. Figure B, panel A plots the equilibrium number of infected cells in the two compartments as a function of 1/b, and the dependence is qualitatively the same compared to the dependence on the CTL expansion rate, c (compare to Figure 2A in main text). Figure B, panel B uses these data to plot the total number of infected cells against the F:EF ratio, and the pattern is again qualitatively the same compared to varying the parameter c (Figure 2B in main text).

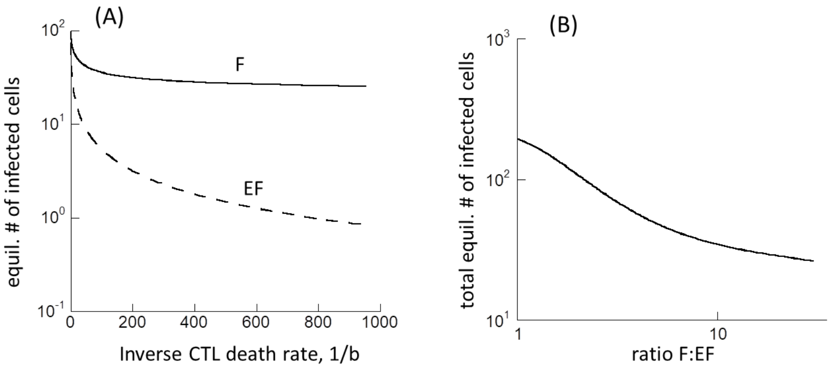

Figure B: (A) Equilibrium number of virus-producing cells in the two compartments as a function of 1/b. F=follicular compartment, EF=extrafollicular compartment. (B) Relationship between the equilibrium number of virus-producing cells and the ratio of follicular to extrafollicular virus load (F:EF). Parameters were the same as in Figure 2A, but with c=5.

Varying the rate of CTL homing to the follicular compartment: In the main part of our analysis, we varied the rate of CTL homing from the extrafollicular to the follicular compartment, g. An increase in the parameter g corresponds to increased homing to the follicular compartment, and hence to more CTL being present in that site. This process is countered by the rate of CTL homing away from the follicular compartment, towards the extrafollicular site, h. If the parameter g is sufficiently large, a decrease in the parameter h has an identical effect compared to an increase in g. Therefore, it is really the ratio of g/h that determines outcome. This is shown in Figure C, comparing the black and red lines. The black line is the same graph

shown in in the main text in Figure 2C, where only the parameter g was varied. The red line represents the outcome of corresponding simulations where the parameter h was varied. For lower values of g, however, we find that a decrease in h leads to different results compared to an equivalent increase in g. Hence, it is not the ratio g/h that determines outcome (Figure C, compare blue and black lines). The reason is that for relatively low values of g, comparatively few CTL are present in the follicular compartment, and the number of cells that can migrate back to the EF compartment becomes limiting.

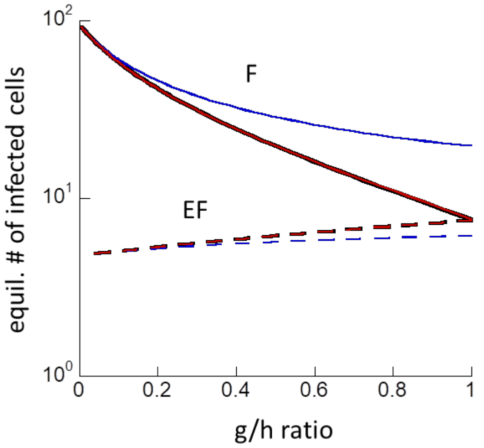

Figure C: Equilibrium number of virus-producing cells in the two compartments as a function of the ratio g/h. The black lines are identical to the ones in Figure 2C, and were generated by varying the parameter g. The red lines were generated by varying the parameter h, assuming a relatively large value of g (g=1). The blue lines were also generated by varying the parameter h, but assuming a lower value of g (g=0.01). The remaining parameter values were the same as in Figure 2C.

Effect of infected cell migration rate: In the computer simulations presented in the main text, a relatively low rate of migration of infected cells between the compartments, η, was assumed. We also explored the outcome when the migration rate of infected cells was increased, to an order of magnitude that is equivalent to the CTL migrations rates that were assumed in those simulations. Results remained qualitatively the same, although the extent of compartmentalization was reduced for larger values of η (Figure D).

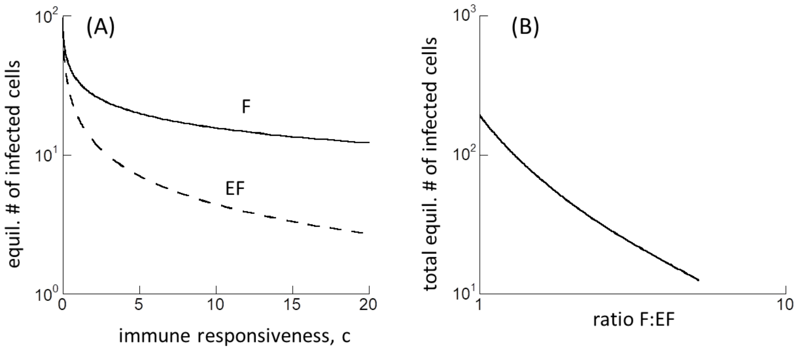

Figure D: Same simulations as in Figure 2A and 2B in the main text, but with a larger infected cell migration rate η=0.5. (A) Equilibrium number of virus-producing cells in the two compartments as a function of the CTL responsiveness, c, which correlates with the strength of the CTL response. F=follicular compartment, EF=extrafollicular compartment. (B) Relationship between the equilibrium number of virus-producing cells and the ratio of follicular to extra-follicular virus load (F:EF).

If the rate of infected cell migration, η, is increased to sufficiently high levels, the degree of compartmentalization becomes very small (Figure E). Effectively, the system behaves more like a well-mixed pool of cells.

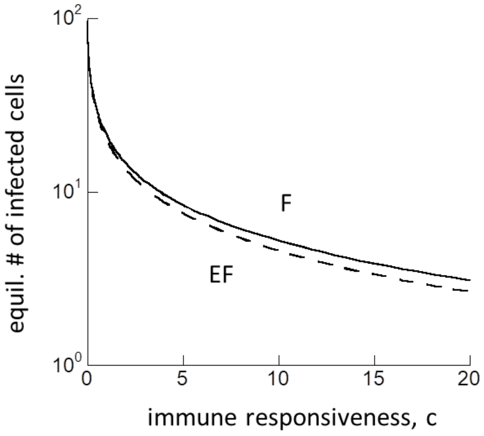

Figure E: Same simulations as in Figure 2A, but with a large infected cell migration rate η=10. The equilibrium number of virus-producing cells in the two compartments is plotted as a function of the CTL responsiveness, c, which correlates with the strength of the CTL response. F=follicular compartment, EF=extrafollicular compartment.

Compartment-specific parameters: So far, all simulations assumed that the target cell kinetics were the same in the two compartments, i.e. that the number of target cells in the absence of infection was the same in the follicular and extrafollicular compartments. Further, it was assumed that infected cells migrated with equal rates in both directions. Here, were repeat the simulations in Figure 2A and B from the main text assuming that target cell numbers are different in the two compartments in the absence of infection, and that migration rates are different in each direction. Thus, we assume that the values of λ_e and λ_f were different. The migration rate of infected cells from the extrafollicular to the follicular compartment is now denoted by η_1,

while the migration rate in the opposite direction is denoted by η_2. As shown in Figure F, the general dependencies remain the same compared to the ones presented in Figure 2A/B in the main text.

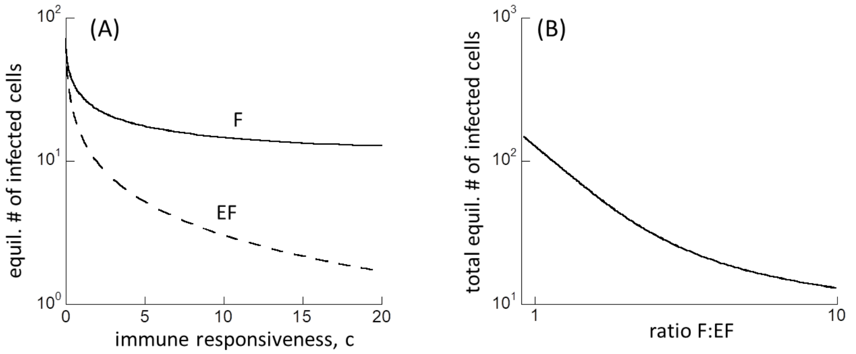

Figure F: Same simulations as in Figure 2A and 2B in the main text, but with λe=50 and λf=30, as well as η1=0.1 and η2=0.01. F=follicular compartment, EF=extrafollicular compartment.

Target cell activation, infection permissiveness, and compartmentalization

While CD4 T cells in the follicular compartment are always activated and by definition highly permissive to infection, in the extra-follicular compartment, target cell permissiveness might depend on antigen-induced stimulation of the T cells [1]. In other words, the permissiveness of the T cells to infection might depend on virus load in the extra-follicular compartment. This could in principle be another mechanism that drives the observed compartmentalization of infected cells, and we tested this hypothesis with a mathematical model. To do so, model (1) in the main text was modified to assume that productive target cell infection requires their activation in the extra-follicular compartment, and that activation occurs in response to antigenic stimulation by the virus. For the dynamics in the follicular compartment, we assumed that all target cells are activated and permissive to infection. Denoting resting (non-susceptible) target

cells in the extra-follicular compartment by W_e, and activated target cells as X_e, the model is thus given by the following set of ordinary differential equations.

$$\dot{W}_e = \xi - fW_e - rW_eY_e; \qquad \dot{X}_e = rWY_e - dX_e - \beta_e X_e Y_e$$

$$\dot{Y}_e = \beta_e X_e Y_e - aY_e - pY_e Z_e - \eta Y_e + \eta Y_f;$$

$$\dot{X}_f = \lambda_f - dX_f - \beta_f X_f Y_f; \qquad \dot{Y}_f = \beta_f X_f Y_f - aY_f - pY_f Z_f - \eta Y_f + \eta Y_e$$

$$\dot{Z}_e = cY_e - bZ_e - gZ_e + hZ_f; \qquad \dot{Z}_f = gZ_e - bZ_f - hZ_f.$$

Resting target cells are produced with a rate ξ, die with a rate f, and become activated by the presence of productively infected cells with a rate r.

In the context of a single compartment, virus dynamics under the assumption that target cell permissiveness depends on virus-induced cell activation has been studied before [2], and the properties are briefly outlined here. This kind of model is characterized by two equilibria being simultaneously stable: the virus extinction equilibrium, and a virus persistence outcome. To which equilibrium the system converges is determined by initial conditions, in particular initial virus load. If initial virus load is relatively high, enough stimulation of target cells occurs to generate a sufficient number of activated cells to enable persistent infection. If initial virus load is low, however, not enough stimulation is present, which fails to generate sufficient numbers of activated target cells for sustained replication. As a consequence, the population of virus-producing cells goes extinct. This extinction is further promoted by the presence CTL responses, which can suppress virus load to a level that is too low to maintain a sufficient level of cell activation. The same kind of dynamics can occur in our current model and we focus parame-

ters in which the virus population is essentially driven extinct in a one-compartment setting due to lack of target cell activation. The model discussed here, however, takes into account a second compartment (follicular compartment), from which infected cells can enter by migration. The infected cells that migrate to the extra-follicular compartment can also contribute to target cell stimulation. Hence, the outcome depends on the rate of infected cell migration from the F to the EF compartment (parameter η), which is described as follows.

If the migration rate, η, is relatively high, the incoming infected cells activate a sufficient number of target cells in the EF compartment such that sustained infection in this compartment is possible (Figure G, panel A). If the migration rate, η, is low, however, this is not the case anymore. In this scenario, no active virus replication occurs in the EF compartment, and only a small population of extra-follicular virus-producing cells remains, as a result of migration from the follicular compartment (Figure G, panel C). For intermediate migration rates, η, dynamics are observed that lie between the two cases described above (Figure G, panel B). The number of extra-follicular virus-

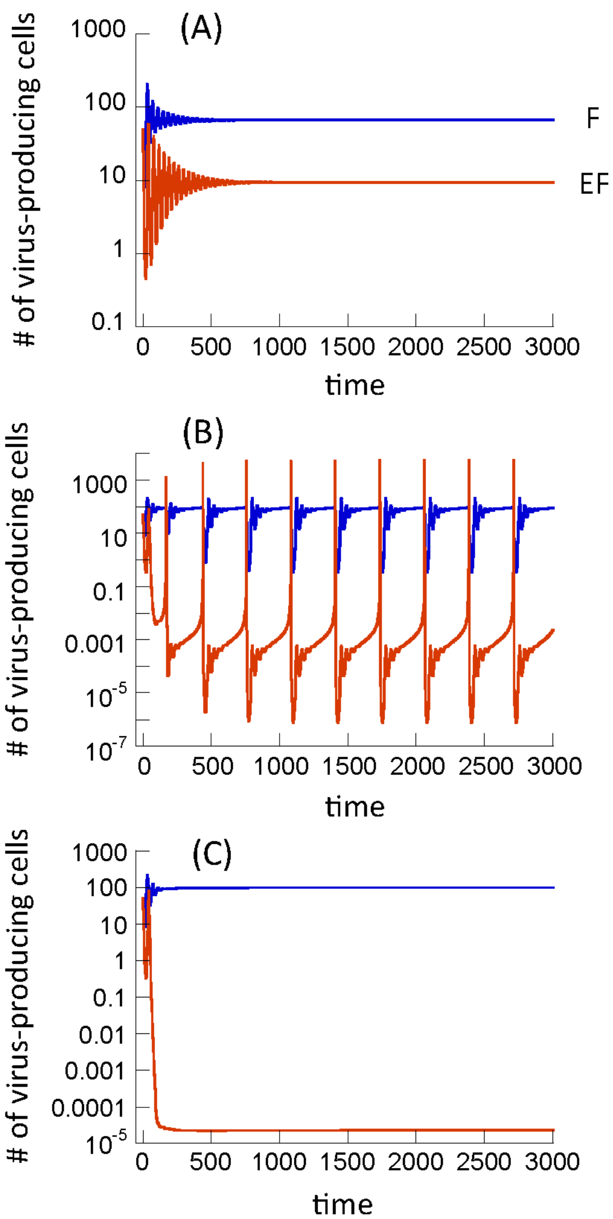

Figure G: *Dynamics in F and EF compartments observed in the target cell activation model. Parameters were chosen as follows. $\xi = \lambda_f = 50$, $f=0.001$, $d=0.01$, $\beta_e =$, $\beta_f = 0.00072$, $a=0.45$, $c_e=2$, $b=0.01$, $p=0.001$, $g=0.5$, $h=2$. The panels differ in the migration rate of virus producing cells, η. (A) $\eta=10^{-3}$, (B) $\eta=10^{-5}$, (C) $\eta=10^{-7}$.*

producing cells declines towards extinction, since CTL push virus loads to levels that are too low to maintain enough activated target cells. Limited migration of virus-producing cells, however, periodically activates target cells, which allows the virus population to grow in the EF compartment and to further activate T cells, resulting in positive feedback-like dynamics and virus expansion (Figure G, panel B). The CTL response, however, reacts to this and eventually pushes the virus population again to levels that are too low to sustain target cell activation. These dynamics are repeated periodically, resulting in stable population cycles over time (Figure G, panel B).

The most relevant regime for our investigation is the one shown in Figure G, panel C, where virus-producing cells in the extra-follicular compartment are not sustained by virus replication in that site, but by influx of cells from the follicular compartment. This results in stronger compartmentalization compared to model (1) in the main text, as shown in Figure H. In contrast to the properties of model (1), however, the target cell activation model fails to predict a rise of virus load in the extra-follicular compartment following CTL depletion (Figure H). The reason is that virus load in the EF compartment is too low to maintain a sufficient number of activated target cells in the presence of CTL. Once the CTL have been removed, there are still not enough ac-

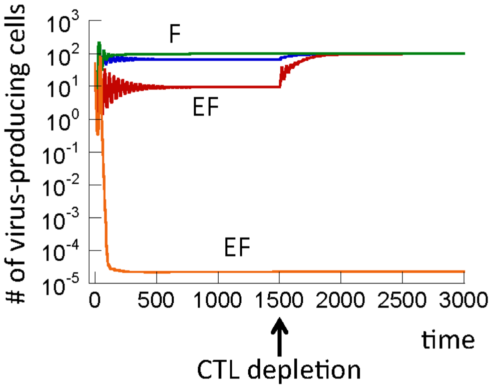

Figure H: *Comparing the pre- and post CTL depletion dynamics in model (1) from the main text (F=blue, EF=red) and in the target cell activation model in the regime depicted in Figure G, panel C (F=green, EF=orange). For the target cell activation model, CTL depletion does not result in an increased virus load. See text for explanation. Parameters were chosen as follows.* $\xi = \lambda_e = \lambda_f = 50$, $f=0.001$, $d=0.01$, $\beta_e=$, $\beta_f=0.00072$, $a=0.45$, $c_e=2$, $b=0.01$, $p=0.001$, $g=0.5$, $h=2$. *For model (1),* $\eta=0.01$. *For the target cell activation model,* $\eta=10^{-7}$.

tivated target cells present to enable virus expansion, because a threshold amount of virus in the EF compartment is required to achieve this. Hence, without a sufficient external influx of target cell stimulation, an immediate growth of the virus population in the EF compartment following CTL depletion is not expected in this model. Therefore, the properties of this scenario are in conflict with the experimental data discussed in the main text.

We now focus on the parameter regime shown in Figure G, panel A, where infected cell influx from the F compartment is sufficient to maintain enough activated target cells to allow persistent virus replication in the EF compartment. We asked whether in this scenario, the requirement of target cell activation for infection changes the compartmentalization dynamics in a significant way. Hence, we compare computer simulations of model (1) with those of the activation model, assuming that the rate of target cell production is identical in the two models (λ_e=ξ). As shown in Figure I, the equilibrium numbers of virus-producing cells in the two compartments are very similar to each other. The reason is that in these types of mathematical models, equilibrium virus load is mostly determined by parameters of the immune response, rather than by parameters that govern the target cell dynamics [3]. Hence, the models suggest that in this parameter region, the requirement of target cell activation for infection does not contribute significantly to the observed compartmentalization of the virus population.

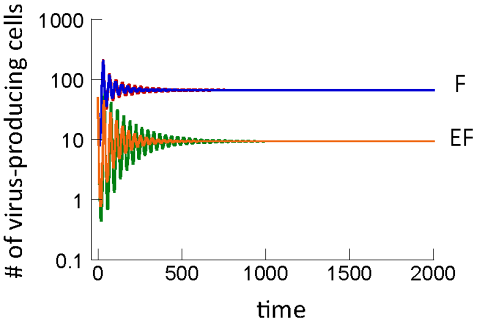

Figure I: Comparison of the dynamics observed in model (1) (red and green) and in the target cell activation model for the regime depicted in Figure G, panel A, where enough cell activation occurs to allow sustained infection in the EF compartment (blue and orange). In this case, there is no significant difference between the two models. Parameters were chosen as follows. *ξ= λ_e =λ_f =50, f=0.001, d=0.01, β_e=, β_f=0.00072, a=0.45, c_e=2, b=0.01, p=0.001, g=0.5, h=2. For model (1), η=0.01. For the target cell activation model, η=10^{-3}.*

Finally, we consider the parameter regime in Figure G, panel B, where persistent population cycles are observed. We are not aware of data that document such cycles in the EF compartment, and hence the biological relevance of this regime is unclear. The occurrence of sustained population cycles is not the consequence of the two-compartment nature of the model. The same kind of dynamics are observed if we consider a single-compartment model with a constant external influx of virus-producing cells. It also continues to be observed if the model is altered in a number of ways, for example by introducing saturation terms into the rate of target cell activation or the rate of infection. The potential meaning of this parameter regime remains to be explored.

1. Cartwright EK, Spicer L, Smith SA, Lee D, Fast R, et al. (2016) CD8(+) Lymphocytes Are Required for Maintaining Viral Suppression in SIV-Infected Macaques Treated with Short-Term Antiretroviral Therapy. Immunity 45: 656-668.
2. Wodarz D, Lloyd AL, Jansen VA, Nowak MA (1999) Dynamics of macrophage and T cell infection by HIV. J Theor Biol 196: 101-113.
3. Wodarz D (2007) Killer Cell Dynamics: mathematical and computational approaches to immunology. New York: Springer.
